# Changes in Serum Levels of NINJ1 and HMGB1 in Children with Kawasaki Disease and Their Clinical Significance

**DOI:** 10.3390/biomedicines14020402

**Published:** 2026-02-10

**Authors:** Tong Tong, Ting Zhao, Jiawen Xu, Fei Liu, Linghao Cai, Xinrui Mao, Chunhong Xie, Yujia Wang, Fangqi Gong

**Affiliations:** 1 Department of Cardiology, Children’s Hospital, Zhejiang University School of Medicine, National Clinical Research Center for Children and Adolescents’ Health and Diseases, No. 3333 Binsheng Road, Hangzhou 310052, China; tongtonggo@zju.edu.cn (T.T.); 11707048@zju.edu.cn (T.Z.); xujiawen@zju.edu.cn (J.X.); xch_hz@hotmail.com (C.X.); 2 Department of Nephrology, Children’s Hospital, Zhejiang University School of Medicine, National Clinical Research Center for Children and Adolescents’ Health and Diseases, Hangzhou 310052, China; feiliu81@zju.edu.cn (F.L.); sherryxr0320@163.com (X.M.); 3 Department of Neonatal Surgery, Children’s Hospital, Zhejiang University School of Medicine National Clinical Research Center for Children and Adolescents’ Health and Diseases, Hangzhou 310052, China; cailinghao@zju.edu.cn

**Keywords:** Kawasaki disease, coronary artery lesions, NINJ1, HMGB1, LDH

## Abstract

**Purpose**: Kawasaki disease (KD) is an acute systemic vasculitis that can result in coronary artery lesions (CALs). This study aims to explore the expression levels of serum Ninjurin-1 (NINJ1) and high-mobility group box 1 (HMGB1) in the acute phase of KD and evaluate their clinical significance. **Methods**: A total of 180 children were enrolled, comprising 113 KD patients, 35 healthy controls (HCs), and 32 febrile controls whose clinical data were collected. Serum levels of NINJ1, HMGB1, Lactate Dehydrogenase (LDH), and routine inflammatory markers were compared across groups. Serum levels of NINJ1 and HMGB1 were measured via ELISA. Correlations were analyzed using Spearman tests. The diagnostic and predictive performance of biomarkers was assessed using Receiver Operating Characteristic (ROC) curve analyses. **Results**: Serum levels of NINJ1 and HMGB1 were significantly elevated in the KD group compared with both the HC and FC groups (all *p* < 0.001). NINJ1 levels were positively correlated with the *z*-scores of coronary arteries and were significantly higher in the CAL subgroup than in the non-CAL subgroup (*p* = 0.004). A strong positive correlation was observed between serum NINJ1 and HMGB1 levels in the KD group (*p* < 0.001). **Conclusions**: Elevated serum NINJ1 levels during the acute phase of KD were associated with the presence of CALs, while HMGB1 shows promise in differentiating KD from other febrile illnesses. These findings collectively suggest that the NINJ1-HMGB1 axis may offer novel insights into the mechanisms underlying KD vasculitis, supporting further investigation into its potential clinical relevance.

## 1. Introduction

Kawasaki disease (KD) is an acute, self-limited systemic vasculitis that predominantly affects infants and young children, and has become the leading cause of acquired heart disease in children across developed countries [1,2]. The most formidable complication of KD is the development of coronary artery lesions (CALs), including coronary artery aneurysms, which can result in long-term cardiovascular morbidity and even sudden death [2,3]. Although the administration of intravenous immunoglobulin (IVIG) has markedly reduced the incidence of CALs, approximately 10–20% of patients exhibit resistance to initial IVIG treatment, leaving them at a high risk for CAL development [2]. Early diagnosis and risk stratification are therefore critical. However, the clinical presentation of KD is non-specific and often mimics other common febrile illnesses. Compounding this challenge, no specific biomarker currently exists that can reliably confirm diagnosis or predict CAL development.

Furthermore, the etiology of KD remains largely unknown, and it is generally believed to involve an abnormal immune and inflammatory response triggered by unknown infectious agents in genetically susceptible individuals [4,5]. The central pathological feature of KD is injury and dysfunction of systemic vascular endothelial cells [6]. Growing evidence suggests that damage-associated molecular patterns (DAMPs) play a pivotal role in the disease’s pathogenesis [7]. DAMPs, also known as alarmins, are intracellular molecules released from damaged or necrotic cells that can activate the innate immune system and propagate sterile inflammation [8].

Ninjurin-1 (NINJ1) is a transmembrane protein that plays a critical role in executing lytic cell death. Upon activation, NINJ1 oligomerizes to form a pore-like structure in the plasma membrane, causing membrane rupture [9,10]. This active process of cell lysis is a final common step in the release of numerous pro-inflammatory intracellular contents, including high-mobility group box 1 (HMGB1) and other DAMPs [11]. HMGB1, a well-characterized DAMP, is elevated in KD and is known to exacerbate endothelial dysfunction and systemic inflammation [12,13]. Therefore, NINJ1 is not simply a passive player but actively amplifies the inflammatory response by controlling the release of potent immunostimulatory signals. The role of NINJ1 in inflammation is gaining recognition across a range of autoimmune and inflammatory conditions. For instance, studies have shown that NINJ1 deficiency or blockade ameliorates disease severity in experimental models by reducing the release of DAMPs and subsequent immune cell infiltration [14,15,16]. This positions NINJ1 as both a promising therapeutic target and a potential biomarker of active, destructive inflammation across multiple diseases. Additionally, serum NINJ1 has been identified as a promising biomarker for systemic lupus erythematosus, aiding in the identification of lupus nephritis and thrombocytopenia and potentially helping with disease stratification and monitoring [17].

Despite its key role in regulating DAMP release and inflammation, the involvement of NINJ1 in KD remains entirely unexplored. To address this gap, we measured the serum levels of NINJ1 and HMGB1—along with Lactate Dehydrogenase (LDH), a widely recognized marker of general cell damage. By exploring the interrelationships and clinical significance of NINJ1, HMGB1, and LDH, our study aims to provide new insights into the pathogenesis of KD and evaluate their potential utility as novel indicators in improving the differential diagnosis of KD and to further assess their role in predicting the risk of CALs.

## 2. Materials and Methods

### 2.1. Subjects

A total of 113 children diagnosed with KD and 35 healthy children (HC) who received routine physical examinations at the Department of Cardiology, Children’s Hospital, Zhejiang University School of Medicine were enrolled in this study. In total, 32 febrile patients were enrolled as febrile controls (FCs), including those with sepsis, bronchopneumonia or pneumonia, and urinary tract infection. For all patients, demographic information was collected, including age, sex, and the disease day at sampling. The day of fever onset was recorded as the first day of the course of the disease course. This study was approved by the Ethics Committee of the Children’s Hospital of Zhejiang University School of Medicine (No. 2021-IRB-320).

### 2.2. Inclusion Criteria

The inclusion criteria for the KD group are sa follows: (1) patients meeting diagnostic criteria for KD; (2) patients newly diagnosed with KD; (3) blood tests conducted during the acute phase of KD, with coronary artery ultrasonography performed before and after intravenous immunoglobulin treatment; (4) complete medical records available.

### 2.3. Exclusion Criteria

The exclusion criteria were as follows: (1) patients with a history of KD or cardiovascular disease; (2) patients with rheumatic or other immune system diseases; (3) patients who received treatment with IVIG, corticosteroids, or immunosuppressants before admission; (4) patients with missing data.

### 2.4. Definition and Subgrouping Criteria

KD patients were classified according to the following definitions [2].

Coronary Artery Lesions (CALs): Following the 2017 AHA guidelines, echocardiographic findings at 4–6 weeks were used to define CALs based on *z*-scores of the coronary arteries. A *z*-score of < 2.0 indicated no involvement; ≥2.0 to <2.5 indicated dilation; and ≥2.5 indicated aneurysm. Patients were subsequently grouped into CAL (*z*-score ≥ 2.0) and non-CAL (*z*-score < 2.0) cohorts.

### 2.5. Study Outcomes

(1) Primary outcomes: The association between serum NINJ1 and coronary involvement, specifically (a) the comparison of NINJ1 levels between CAL and non-CAL groups, (b) the correlation between NINJ1 levels and the *z*-score of coronary arteries, (c) the correlation between NINJ1 and HMGB1 levels, and (d) the difference in NINJ1 and HMGB1 levels between KD patients and FC.
(2) Secondary outcomes: This includes all other group comparisons, correlation analyses with inflammatory markers, diagnostic performance for KD differentiation, and the predictive Receiver Operating Characteristic (ROC) curve analyses for CALs.

### 2.6. Sample Collection and Measurement of NINJ1 and HMGB1

Serum samples were collected from participants with KD before infusion of IVIG. All samples were stored at −80 °C until testing. All hematological parameters, biochemical indices, and inflammatory cytokines were measured using standard clinical automated analyzers and their corresponding reagents in the hospital laboratory department. The concentration of NINJ1 (RX105287H, Ruixinbio, Quanzhou, China) and HMGB1 RH105888H, Ruixinbio, Quanzhou, China) was measured with an enzyme-linked immunosorbent assay (ELISA) according to the manufacturer’s instructions. Their clinical data, including age, gender, inflammatory markers, and echocardiographic outcomes, were retrieved and collected retrospectively from electronic medical records.

### 2.7. Statistical Analysis

Statistical analysis was performed using SPSS 27.0, GraphPad Prism 10 software, and R programming language (version 4.2.1). Normality of continuous variables was assessed using the Shapiro–Wilk test. Normally distributed data were represented by $\bar{x}$ ± s and compared between two groups with the independent sample *t*-tests. Nonnormally distributed quantitative data were presented as a median (Q1 and Q3) and compared using the Mann–Whitney U test. Categorical data, presented as *n* (%), were compared using the χ^2^ test or Fisher’s exact test as appropriate. Correlation analyses were conducted to evaluate the relationships between serum NINJ1, HMGB1, and LDH levels; inflammatory markers; and coronary artery *z*-scores. Spearman’s rank correlation analysis was employed, with the correlation coefficient reported as r. Linear regression was used to establish a quantitative relationship model between serum NINJ1 levels and coronary artery *z*-scores, thereby quantifying the predictive effect. Given the limited number of CAL events, multivariable analysis was performed using Firth’s penalized maximum likelihood logistic regression to mitigate bias. The model included the following covariates selected a priori based on clinical relevance: serum NINJ1 level, age, gender, ALB, PLT, and Hb. The diagnostic and predictive performance of serum NINJ1, HMGB1, LDH, and other inflammatory markers was evaluated using ROC curve analysis. The optimal cut-off values for biomarkers in ROC curve analyses were determined by maximizing the Youden index (J = sensitivity + specificity − 1). The Area Under the Curve (AUC), along with its 95% Confidence Interval (CI), was calculated for each biomarker. A statistical comparison of the AUCs was performed using the DeLong test, a non-parametric method for comparing correlated ROC curves derived from the same sample. A *p*-value < 0.05 was considered statistically significant. To control the false discovery rate (FDR) in the correlation analyses between NINJ1 and inflammatory markers, *p*-values were adjusted using the Benjamini–Hochberg procedure, yielding q-values. A q-value < 0.05 was considered significant for exploratory tests.

## 3. Result

### 3.1. Demographic Information and Inflammatory Markers in the KD, FC, and HC Groups

**Figure 1:**
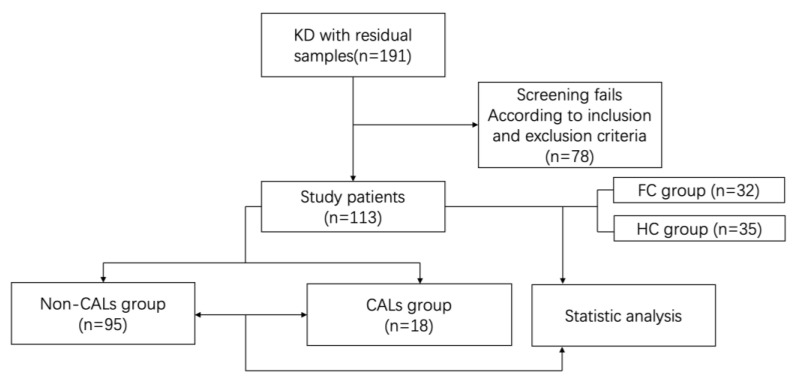
Flowchart of the study.

The general characteristics and laboratory parameters of the study participants are summarized in Table 1. A total of 180 children were enrolled between 1 October 2023 and 30 January 2025 comprising 113 in the KD group, 32 in the FC group, and 35 in the HC group (Figure 1). Compared with the HC group, children with KD were younger (*p* < 0.01), while there was no significant age difference observed between KD and FC patients. No significant difference in gender distribution was observed among the three groups (*p* > 0.05).

In terms of inflammatory markers, KD patients exhibited significant systemic inflammation compared with the HC group. This was manifested by markedly elevated levels of white blood cells (WBCs) (*p* < 0.01), monocytes (Mono) (*p* < 0.01), lymphocytes (*p* < 0.05), platelets (PLTs) (*p* < 0.01), C-reactive protein (CRP) (*p* < 0.01), alanine aminotransferase (ALT) (*p* < 0.01), and aspartate aminotransferase (AST) (*p* < 0.05), while hemoglobin (Hb) (*p* < 0.01) and albumin (ALB) (*p* < 0.01) levels were significantly reduced (Table 1). Compared with the FC group, ESR (*p* < 0.001), ALT (*p* < 0.001), and AST (*p* < 0.01) levels were significantly higher in KD patients, while Hb (*p* < 0.05) and sodium (Na) (*p* < 0.01) levels were significantly reduced in the KD group (*p* < 0.001) (Table 1).

### 3.2. Serum Levels of NINJ1, HMGB1, and LDH Were Significantly Higher in the KD Group Compared with the FC and HC Groups

**Figure 2:**
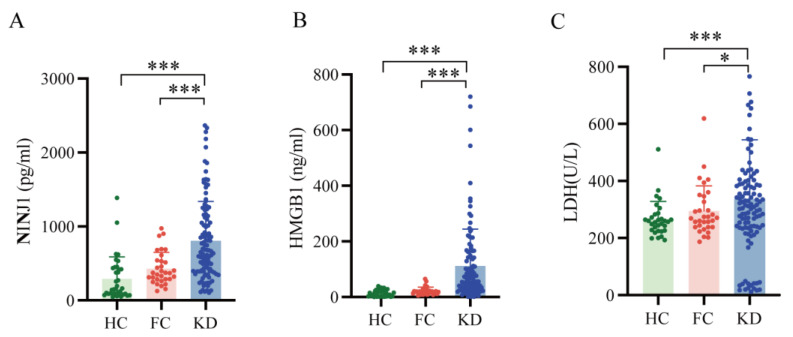
Serum levels of NINJ1, HMGB1 and LDH were significantly higher in KD group compared with HC group and FC group. (**A**) Compared to the HC (156.90 pg/mL) and FC (370.98 pg/mL) groups, the KD group (665.87 pg/mL) showed substantially elevated median serum levels of NINJ1 (*p* < 0.001). (**B**) Compared to the HC (8.28 ng/mL) and FC (19.61 ng/mL) groups, the KD group (55.52 ng/mL) showed substantially elevated median serum levels of HMGB1 (*p* < 0.001). (**C**) Compared to the HC (257 ng/mL) and FC (270 ng/mL) groups, the KD group (322.5 ng/mL) showed substantially elevated median serum levels of LDH (*p* < 0.001 and *p* < 0.05, respectively). NINJ1, Ninjurin-1; HMGB1, High Mobility Group Box 1; LDH, Lactate Dehydrogenase; KD, Kawasaki Disease; HC, healthy control; FC febrile control. * *p* < 0.05; *** *p* < 0.001.

Serum concentrations of NINJ1, HMGB1, and LDH were compared across the study groups, and their levels were significantly higher in patients with the KD compared to both the HC and the FC group. Compared to the HC and FC groups, the KD group showed substantially elevated median serum levels of NINJ1 (665.87 vs. 156.90 and 370.98 pg/mL, *p* < 0.001), HMGB1 (55.52 vs. 8.28 and 19.61 ng/mL, *p* < 0.001), and LDH (322.5 vs. 257 U/L, *p* < 0.001 and 270 U/L, *p* < 0.05) (Table 1 and Figure 2). This finding directly confirms that NINJ1 and HMGB1—two molecules associated with cellular injury—are substantially released into the blood during the acute phase of KD. This pattern aligns with the trend observed in LDH, a classic enzymatic biomarker representing tissue cell damage, collectively indicating severe cellular injury and necrosis occurring during the pathological process of KD. The fact that NINJ1 and HMGB1 levels in KD patients were significantly higher not only in HC but also in patients with other febrile illnesses suggests that these markers may offer enhanced specificity in distinguishing KD from common infectious febrile conditions.

### 3.3. Correlation Analysis of Serum NINJ1

**Figure 3:**
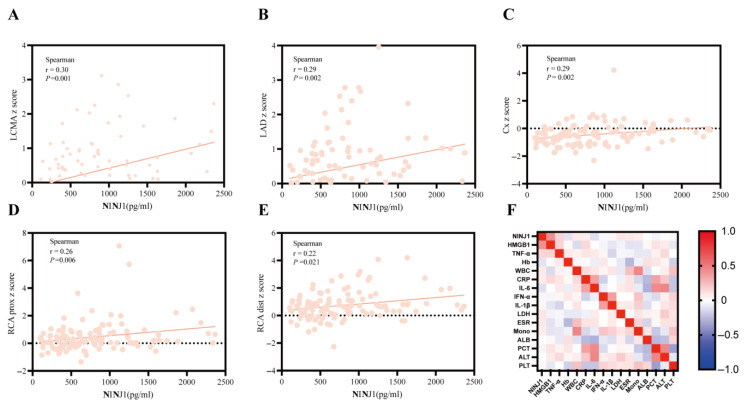
Correlation analysis of serum NINJ1 levels in KD patients. (**A**) Serum NINJ1 levels showed significant positive correlations with *z*-scores in the left main coronary artery (LMCA) (r = 0.30, *p* = 0.001); (**B**) Serum NINJ1 levels showed significant positive correlations with *z*-scores in the left anterior descending (LAD) artery (r = 0.29, *p* = 0.002); (**C**) Serum NINJ1 levels showed significant positive correlations with *z*-scores in the circumflex (Cx) artery (r = 0.29, *p* = 0.002); (**D**) Serum NINJ1 levels showed significant positive correlations with *z*-scores in the right coronary artery proximal (RCA prox) (r = 0.26, *p* = 0.006); (**E**) Serum NINJ1 levels showed significant positive correlations with *z*-scores in the right coronary artery distal (RCA dist) (r = 0.22, *p* = 0.021); (**F**) Association of NINJ1 Levels with laboratory parameters. NINJ1, Ninjurin-1; LMCA, Left Main Coronary Artery; LAD, left anterior descending; Cx, circumflex; RCA prox, Right Coronary Artery proximal; RCA dist, Right Coronary Artery distal; HMGB1, High Mobility Group Box 1; LDH, Lactate Dehydrogenase; WBC, White blood cell; Hb, Hemoglobin; CRP, C-reactive protein; PCT, procalcitonin; IL-1β, Interleukin-1β; IL-6, Interleukin-6; TNF-α, Tumor Necrosis Factor-alpha; ALB, albumin; ALT, Aminotransferase; ESR, erythrocyte sedimentation rate.

Serum NINJ1 levels showed significant positive correlations with *z*-scores in the left main coronary artery (LMCA) (r = 0.30, *p* = 0.001, Figure 3A), left anterior descending (LAD) artery (r = 0.29, *p* = 0.002, Figure 3B), circumflex (Cx) artery (r = 0.29, *p* = 0.002, Figure 3C), right coronary artery proximal (RCA prox) (r = 0.26, *p* = 0.006, Figure 3D), and right coronary artery distal (RCA dist) (r = 0.22, *p* = 0.021, Figure 3E). This finding revealed an association between NINJ1 and the severity of injury in specific coronary artery branches for the first time, suggesting that NINJ1 not only participates in systemic inflammation but may also play a crucial role in the pathophysiology of local coronary inflammation and injury. Spearman’s correlation analysis further revealed that NINJ1 levels showed significant positive correlations with HMGB1 (*p* < 0.01) (Figure 3F). However, our study showed a poor correlation between NINJ1 and other laboratory characteristics. Moreover, no significant correlations were found between disease day and NINJ1 or HMGB1 levels (*p* = 0.53 and *p* = 0.58; Supplementary Figure S1). After FDR correction (q-value), the correlation between NINJ1 and HMGB1 remained significant (q < 0.001, Supplementary Table S1).

### 3.4. Difference in Demographic Information and Inflammatory Markers Between CAL and nCAL Groups

The median disease day was 5.00 days (IQR 4.00–6.00) in the total population, 5.00 days (IQR 4.00–6.00) in the nCAL group, and 5.00 days (IQR 4.00–6.75) in the CAL group, with no statistically significant difference between the groups (*p* = 0.713). KD patients with CALs were younger (*p* = 0.013) and exhibited more severe inflammatory and cellular injury states. Compared with the nCAL group, the CAL group showed significantly higher levels of Mono counts (*p* = 0.042), PLT (*p* = 0.006), and interferon-α (IFN-α) (*p* = 0.041), while Hb (*p* = 0.028) was significantly lower (Table 2). This indicates that CAL development is associated with stronger immune activation.

### 3.5. Serum Levels of NINJ1 and LDH Were Significantly Higher in the CAL Group Compared with the nCAL Group in KD

**Figure 4:**
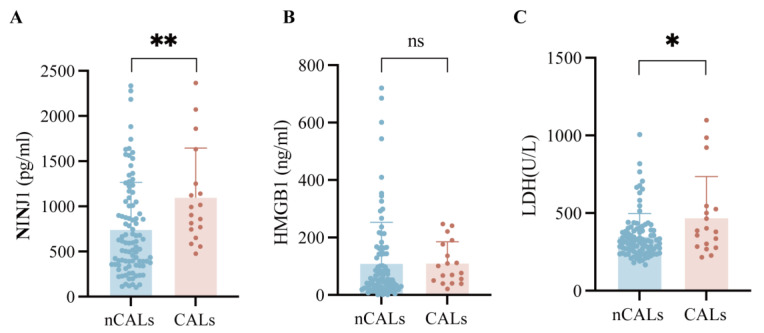
The serum levels of NINJ1, HMGB1 and LDH in CALs patients (**A**) The level of NINJ1 is significantly higher in CALs group (948.34 pg/mL vs. 596.84 pg/mL, *p* = 0.004). (**B**) No significant difference in serum HMGB1 levels was observed between the CALs group and the nCALs group (88.48 ng/mL vs. 52.36 ng/mL, *p* = 0.102). (**C**) The level of LDH is significantly higher in KD patients (383 U/L vs. 315.5 U/L, *p* = 0.049). NINJ1, Ninjurin-1; HMGB1, High Mobility Group Box 1; LDH, Lactate Dehydrogenase; KD, Kawasaki Disease; HC, healthy control; CALs, coronary artery lesions. * *p* < 0.05; ** *p* < 0.01; ns, not significant.

Compared with the nCAL group, serum NINJ1 (948.34 pg/mL vs. 596.84 pg/mL, *p* = 0.004) and LDH (383 U/L vs. 315.5 U/L, *p* = 0.049) levels were significantly elevated in the CAL group, while no statistically significant difference in HMGB1 (88.48 ng/mL vs. 52.36 ng/mL, *p* = 0.102) was observed between the two groups (Table 2 and Figure 4). This further confirms that NINJ1 and LDH, as key markers of cellular injury, are specifically associated with the occurrence of coronary artery complications in KD. To adjust for potential confounders and address the issue of low event rates, a multivariable analysis was performed using Firth’s penalized logistic regression. After adjusting for age, gender, ALB, PLT, and Hb, serum NINJ1 levels were not independently associated with the development of CALs (adjusted odds ratio [aOR] = 1.001 per pg/mL, 95% CI: 0.999–1.002, *p* = 0.255). In this model, lower serum ALB levels (aOR = 0.826 per g/L, 95% CI: 0.689–0.967, *p* = 0.016) and higher PLT counts (aOR = 1.006 per 10^9^/L, 95% CI: 1.000–1.012, *p* = 0.038) emerged as significant independent factors associated with an increased risk of CALs (Table 3).

### 3.6. Diagnostic Performance of NINJ1 and HMGB1 for Discriminating KD from FC

**Figure 5:**
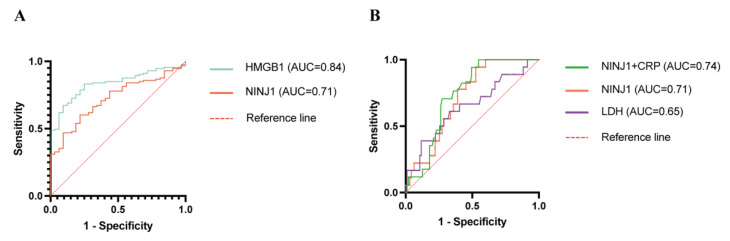
ROC curve of biomarkers. (**A**) Diagnostic performance of NINJ1 and HMGB1 for discriminating KD from FC; (**B**) Predictive value of serum NINJ1, LDH and NINJ1 combined with CRP for KD with CALs. NINJ1, Ninjurin-1; HMGB1, High Mobility Group Box 1; LDH, Lactate Dehydrogenase; KD, Kawasaki Disease; CALs, coronary artery lesions.

To evaluate the potential utility of specific biomarkers in differentiating KD from FC, we assessed the diagnostic accuracy of various serum markers using ROC curve analysis. HMGB1 exhibited a high diagnostic accuracy, with an AUC of 0.84 (95% CI: 0.77–0.90). At a cutoff value of 22.70 ng/mL, HMGB1 achieved a sensitivity of 75% and a specificity of 83%. NINJ1 demonstrated a moderate diagnostic performance, with an AUC of 0.71 (95% CI: 0.63–0.80) (Table 4 and Figure 5A). These results suggest that HMGB1 might be a potential biomarker in distinguishing KD from other febrile illnesses, with balanced sensitivity and specificity.

### 3.7. Predictive Value of Serum NINJ1 and HMGB1 for KD with CALs

Analyses revealed that among the evaluated individual biomarkers, NINJ1 and LDH demonstrated relatively optimal predictive performance (NINJ1, AUC 0.71, 95% CI: 0.61–0.82; LDH, AUC 0.65, 95% CI: 0.50–0.80) (Table 5 and Figure 5B). Notably, at the optimal cutoff value (553.213 pg/mL), NINJ1 achieved high specificity (0.94) despite its moderate sensitivity (0.47), indicating its distinct value in identifying low-risk patients who are unlikely to develop CAL. HMGB1 demonstrated slightly inferior predictive capabilities (AUC = 0.62) (Table 5), limiting its potential for standalone use.

In addition, the combined NINJ1 + CRP model yielded an AUC of 0.74. However, the DeLong test revealed that the difference in AUC between the combined model (AUC = 0.74) and NINJ1 alone (AUC = 0.71) was not statistically significant (*p* = 0.32) (Table 5).

## 4. Discussion

Exploring the key molecules involved in the pathogenesis of KD and identifying reliable biomarkers that can distinguish KD from other febrile illnesses and predict CALs early remain major challenges in KD research. This study not only retrospectively examined the predictive value of NINJ1, HMGB1, and LDH for CALs in KD but also demonstrated their diagnostic value in KD. This is the first study to systematically evaluate the expression level of serum NINJ1 in children with KD and to investigate its association with the known DAMP molecule HMGB1, the cell damage marker LDH, systemic inflammatory status, and coronary artery damage, aiming to provide new experimental insights and clinical tools for earlier recognition, risk stratification, and a better understanding of the pathogenesis of KD.

Our study confirmed that serum levels of NINJ1, HMGB1, and LDH were significantly elevated in KD patients during the acute phase compared to HCs. This finding carries important implications for understanding the pathophysiology of the disease. NINJ1 is the pivotal executor of inflammasome-induced plasma membrane rupture in various forms of non-apoptotic programmed cell death. While this process is essential for clearing damaged or redundant cells, its loss of control can result in the excessive release of DAMPs from cells, thus contributing to a variety of diseases [18]. HMGB1—a key DAMP molecule that is released when cells undergo necrosis or immune cells are activated—strongly activates the innate immune system and promotes the release of pro-inflammatory cytokines, acting as a significant intermediate in cell injury and subsequent amplification of the activities of the immune cascade [19]. LDH, a well-established marker of cellular injury and necrosis, indicates widespread tissue and cellular damage occurring during the acute phase of KD. Our study also uncovered a significant positive correlation between NINJ1 and HMGB1 levels, suggesting that these two molecules may interact synergistically in the pathogenesis of KD.

Another core finding of this study was the association between NINJ1 and CALs. First, serum NINJ1 and LDH levels were markedly elevated in the CAL group compared to the non-CAL group. More significantly, we observed a positive correlation between serum NINJ1 levels and *z*-scores in the LMCA, LAD artery, Cx artery, RCA prox, and RCA dist in KD patients. These results consistently suggest that higher NINJ1 levels are linked to more severe coronary inflammation and damage, suggesting that the high expression of NINJ1 and the release of HMGB1 may cause coronary artery endothelial damage, and they are closely related to coronary endothelial dysfunction and vessel wall lesions. A recent study found that NINJ1, which was highly expressed in macrophages within abdominal aortic aneurysm (AAA) lesions, facilitated AAA formation by promoting the infiltration of immune cells into the aortic wall, providing further evidence for understanding the role of NINJ1 in vascular lesions [20]. Notably, in the multivariable analysis, serum NINJ1 was not an independent predictor of CALs when traditional markers of inflammation and disease severity—including ALB and PLT—were incorporated concurrently. This finding indicates that the significant elevation of NINJ1 is a prominent feature of the acute phase of KD, particularly in severe phenotypes characterized by hypoalbuminemia and thrombocytosis. Thus, its association with CALs may primarily reflect its role as a biomarker linked to the systemic inflammatory-thrombotic state and vascular hyperpermeability, rather than directly indicating an independent pathogenic role in coronary artery injury.

Additionally, we found that the monocyte count was significantly elevated in patients with CALs compared to those without. Monocytes are a vital part of the innate immune system. Under the influence of inflammatory signals such as HMGB1, they can infiltrate the vascular wall and differentiate into macrophages. Activated macrophages are known to be major producers of multiple pro-inflammatory cytokines, playing a central role in the vasculitis pathology of KD [21,22,23]. Recent research also suggested that NINJ1 was upregulated in activated myeloid cells and mediated their programmed membrane rupture [24]. Therefore, we hypothesize that in KD, activated monocytes or macrophages might be an important cellular source of NINJ1 in this pathological process. NINJ1-triggered oligomerization and subsequent plasma membrane rupture in these cells would lead to the release of DAMPs such as HMGB1, which could subsequently promote the further activation of immune cells through a potential positive-feedback loop. This process may play a significant role in the development and progression of CALs in KD. This hypothesis offers a new perspective for understanding the immunopathological mechanisms of KD, yet each step of the proposed causal pathway requires further investigation for validation, particularly through experimental studies.

On the other hand, our study offers dual clinical implications. For differential diagnosis, HMGB1 (AUC = 0.84) demonstrated superior performance over conventional inflammatory markers, with its high sensitivity and specificity providing a promising novel biomarker for the differentiation of KD from other febrile illnesses. Regarding predictive efficacy for CALs, NINJ1 alone demonstrated an AUC value of 0.71 with a high specificity of 94%, indicating its reliability in identifying non-CAL patients.

This study also has some limitations. First, it is a single-center, cross-sectional study with a relatively limited sample size of CAL events (*n* = 18). This limited event rate reduces statistical power and may lead to instability in the performance metrics (such as AUC, sensitivity, specificity, and optimal cutoff values) derived from ROC curve analyses. Therefore, conclusions regarding predictive performance should be considered preliminary and exploratory, requiring validation in larger, prospective cohorts. Moreover, blood samples from KD patients and HC children were obtained under non-strict fasting conditions, which may introduce some bias. Secondly, we only detected the total NINJ1 level in serum, and its specific cellular source remains unclear. Finally, it should be noted that observational correlation analysis itself cannot establish causal relationships, which is another major limitation of this study.

The findings of this study provide future research directions for further exploration. First, the diagnostic and predictive value of NINJ1 and HMGB1 should be verified in larger-scale prospective cohorts to improve the reliability and generalizability of the conclusions. Second, the pathogenic role of NINJ1 in the development of vasculitis should be directly validated by regulating its expression in KD mouse models or cell co-culture systems. Third, techniques such as immunofluorescence and flow cytometry should be employed to accurately localize NINJ1-expressing cell types in pathological specimens from KD patients or animal models, thereby more strongly confirming its association with macrophage infiltration.

In summary, this study is the first to establish that serum NINJ1 is associated with the presence of CALs and closely associated with HMGB1 levels. HMGB1 shows promise as a differential diagnostic biomarker, and NINJ1 holds potential for predicting CALs. These findings not only deepen the understanding of the immunopathological mechanisms of KD but also provide novel molecular targets and insights for developing more precise diagnostic and risk-prediction tools. Future research should focus on validating their clinical utility and elucidating their precise functional roles in vasculitis pathogenesis.

## 5. Conclusions

Elevated serum NINJ1 levels during the acute phase of KD are associated with the presence of coronary artery lesions, while HMGB1 shows promise in differentiating KD from other febrile illnesses. These findings collectively suggest that the NINJ1-HMGB1 axis may offer novel insights into the mechanisms underlying KD vasculitis, supporting further investigation into its potential clinical relevance.

## Figures and Tables

**Table 1:** Comparison of the general data and inflammatory indices of children in KD group and HC group.

Variables	HC (*n* = 35)	KD (*n* = 113)	FC (*n* = 32)	*p* Value
WBC (×10^9^), M (Q_1_, Q_3_)	7.10 (6.31, 8.22)	13.41 (11.27, 16.04) #***	16.14 (10.22, 21.61)	<0.001
Neutrophils (×10^9^), M (Q_1_, Q_3_)	2.90 (2.25, 3.83)	9.33 (6.27, 12.16) ***	10.18 (6.91, 14.17)	<0.001
Monocyte (×10^9^), M (Q_1_, Q_3_)	0.41 (0.35, 0.46)	0.75 (0.56, 1.05) ###***	1.33 (0.85, 1.46)	<0.001
Lymphocyte (×10^9^), M (Q_1_, Q_3_)	3.39 (2.87, 4.39)	2.79 (1.59, 3.95) #*	3.67 (2.08, 5.68)	0.009
PLT (×10^12^), M (Q_1_, Q_3_)	302.00 (246.50, 339.00)	336.00 (271.50, 381.75) *	299.00 (247.50, 368.00)	0.034
CRP (mg/L), M (Q_1_, Q_3_)	0.26 (0.20, 0.57)	61.25 (42.06, 91.01) ***	77.63 (41.95, 106.78)	<0.001
Hb (g/L), Mean ± SD	128.86 ± 7.22	111.06 ± 10.02 #***	116.72 ± 14.54	<0.001
ESR (mm/h), M (Q_1_, Q_3_)	NA	59.25 (42.56, 75.82) ###	27.01 (18.18, 39.60)	<0.001
Na (mmol/L), M (Q_1_, Q_3_)	NA	134.00 (132.00, 136.00) ##	136.50 (134.00, 138.00)	0.002
PCT (ng/mL), M (Q_1_, Q_3_)	NA	0.66 (0.26, 1.76)	0.52 (0.17, 2.67)	0.692
ALB (g/L), M (Q_1_, Q_3_)	44.50 (42.80, 45.70)	38.00 (36.10, 39.80) ***	38.90 (37.25, 40.90)	<0.001
ALT (U/L), M (Q_1_, Q_3_)	14.00 (13.00, 16.50)	28.00 (14.00, 82.00) ###***	13.00 (9.00, 18.00)	<0.001
AST (U/L), M (Q_1_, Q_3_)	32.00 (29.00, 37.00)	37.00 (29.00, 58.00) ##*	28.50 (23.75, 36.50)	0.004
NINJ1 (pg/mL), M (Q_1_, Q_3_)	156.90 (88.56, 427.92)	665.87 (391.75, 1095.13) ###***	370.98 (286.29, 532.88)	<0.001
HMGB1 (ng/mL), M (Q_1_, Q_3_)	8.28 (2.15, 22.48)	55.52 (32.34, 143.44) ###***	19.61 (12.97, 23.23)	<0.001
LDH (U/L), M (Q_1_, Q_3_)	257.00 (227.00, 277.50)	322.50 (266.50, 402.25) #***	270.00 (238.00, 331.75)	<0.001
IL-1β (ng/mL), M (Q_1_, Q_3_)	NA	5.30 (3.40, 6.80)	4.70 (4.20, 6.50)	0.714
IL-6 (ng/mL), M (Q_1_, Q_3_)	NA	63.50 (32.05, 113.50)	53.80 (25.75, 95.20)	0.421
TNF-α (ng/mL), M (Q_1_, Q_3_)	NA	2.30 (1.70, 2.90)	2.00 (1.55, 2.85)	0.682
IFN-α (ng/mL), M (Q_1_, Q_3_)	NA	1.60 (1.08, 2.30)	2.25 (1.75, 2.65)	0.093
Age (month), M (Q_1_, Q_3_)	52.00 (28.00, 70.50)	28.00 (16.00, 52.00) ***	38.00 (10.75, 63.75)	0.001
Gender, n (%)				0.431
F	10 (28.57)	40 (35.40)	14 (43.75)	
M	25 (71.43)	73 (64.60)	18 (56.25)	

HC, healthy control; KD, Kawasaki Disease; WBC, White blood cell; PLT, platelet; CRP, C-reactive protein; Hb, Hemoglobin; ESR, Erythrocyte Sedimentation Rate; PCT, procalcitonin; ALB, Albumin; ALT, Aminotransferase; AST, Aspartate Aminotransferase; NINJ1, Ninjurin-1; HMGB1, High Mobility Group Box 1; LDH, Lactate Dehydrogenase; IL-1β, Interleukin-1β; IL-6, Interleukin-6; TNF-α, Tumor Necrosis Factor-alpha; INF-α, Interferon-alpha; NA, not applicable; * *p* < 0.05, *** *p* < 0.001 versus HC; # *p* < 0.05, ## *p* < 0.01, ### *p* < 0.001 versus FC.

**Table 2:** Comparison of the general data and inflammatory indices of children in the CALs group and nCALs group.

Variables	nCALs (*n* = 95)	CALs (*n* = 18)	Statistic	*p* Value
WBC (×10^9^), M (Q_1_, Q_3_)	13.41 (11.08, 15.86)	13.58 (12.29, 16.77)	Z = −0.78	0.437
Neutrophils (×10^9^), M (Q_1_, Q_3_)	9.18 (6.36, 12.12)	9.63 (6.25, 12.16)	Z = −0.29	0.771
Monocyte (×10^9^), M (Q_1_, Q_3_)	0.71 (0.55, 0.98)	0.88 (0.72, 1.31)	Z = −2.04	0.042 *
Hb (g/L), Mean ± SD	111.94 ± 10.10	106.18 ± 8.17	*t* = 2.22	0.028 *
PLT (×10^12^), M (Q_1_, Q_3_)	331.00 (270.00, 371.00)	396.00 (363.00, 464.00)	Z = −2.74	0.006 **
CRP (mg/L), M (Q_1_, Q_3_)	61.35 (42.05, 92.15)	52.59 (43.16, 86.62)	Z = −0.51	0.607
PCT (ng/mL), M (Q_1_, Q_3_)	0.77 (0.26, 1.87)	0.42 (0.27, 0.80)	Z = −0.65	0.515
ESR (mm/h), M (Q_1_, Q_3_)	55.52 (42.44, 73.05)	66.01 (42.56, 85.11)	Z = −1.08	0.281
ALB (g/L), M (Q_1_, Q_3_)	38.00 (36.50, 39.80)	37.00 (33.00, 39.65)	Z = −1.29	0.197
ALT (U/L), M (Q_1_, Q_3_)	24.00 (14.00, 94.50)	40.00 (11.25, 56.75)	Z = −0.60	0.546
IL-1β (ng/mL), M (Q_1_, Q_3_)	5.10 (3.15, 6.60)	6.40 (5.25, 7.45)	Z = −1.81	0.07
IL-6 (ng/mL), M (Q_1_, Q_3_)	63.50 (31.10, 113.50)	63.25 (40.02, 115.38)	Z = −0.16	0.876
TNF-α (ng/mL), M (Q_1_, Q_3_)	2.30 (1.80, 2.90)	2.35 (1.20, 2.92)	Z = −0.47	0.642
IFN-α (ng/mL), M (Q_1_, Q_3_)	1.55 (1.00, 2.27)	2.25 (1.60, 2.58)	Z = −2.05	0.041 *
NINJ1 (pg/mL), M (Q_1_, Q_3_)	596.84 (356.93, 1029.84)	948.34 (751.65, 1223.79)	Z = −2.87	0.004 **
HMGB1 (ng/mL), M (Q_1_, Q_3_)	52.36 (27.14, 142.83)	88.48 (51.86, 166.16)	Z = −1.64	0.102
LDH (U/L), M (Q_1_, Q_3_)	315.50 (254.25, 387.25)	383.00 (288.50, 519.50)	Z = −1.97	0.049 *
Disease-day (day), M (Q_1_, Q_3_)	5 (4, 6)	5 (4, 6.75)	Z = −0.37	0.713
Age (month), M (Q_1_, Q_3_)	29.00 (19.00, 56.50)	14.50 (12.00, 32.50)	Z = −2.48	0.013 *
Sex, n (%)			χ^2^ = 0.00	0.842
F	34 (35.79)	6 (33.33)		
M	61 (64.21)	12 (66.67)		

WBC, White blood cell; Hb, Hemoglobin; PLT, platelet; CRP, C-reactive protein; PCT, procalcitonin; ESR, Erythrocyte Sedimentation Rate; ALB, Albumin; ALT, Aminotransferase; IL-1β, Interleukin-1β; IL-6, Interleukin-6; TNF-α, Tumor Necrosis Factor-alpha; IFN-α, Interferon-alpha; NINJ1, Ninjurin-1; HMGB1, High Mobility Group Box 1; LDH, Lactate Dehydrogenase; nCALs, non-coronary artery lesions; CALs, coronary artery lesions; * *p* < 0.05; ** *p* < 0.01.

**Table 3:** Factors associated with coronary artery lesions (CALs) in patients with Kawasaki disease: Firth’s penalized multivariable logistic regression analysis.

Variable	Level/Unit	Adjusted Odds Ratio (aOR)	95% Confidence Interval	*p* Value
NINJ1	per 1 pg/mL increase	1.001	(0.999, 1.002)	0.255
Age	per 1 month increase	0.989	(0.958, 1.015)	0.427
Sex	Male vs. Female	1.277	(0.397, 4.623)	0.689
ALB	per 1 g/L increase	0.826	(0.689, 0.967)	0.016 *
PLT	per 10^9^/L increase	1.006	(1.000, 1.012)	0.038 *
Hb	per 1 g/L increase	0.973	(0.905, 1.042)	0.436
Constant (Intercept)	-	449.5	(0.090, 3.12 × 10^6^)	0.158

This model was fitted using Firth’s penalized maximum likelihood method to reduce bias due to the limited number of CALs events (*n* = 18); CALs, coronary artery lesions; KD, Kawasaki Disease; NINJ1, Ninjurin-1; CRP, C-reactive protein; ALB, Albumin; PLT, Platelet count; Hb, Hemoglobin; OR, odds ratio; CI, confidence interval; * *p* < 0.05.

**Table 4:** Diagnostic performance of biomarkers for discriminating Kawasaki Disease (KD) from febrile control (FC).

Biomarker	AUC (95% CI)	Sensitivity	Specificity	Cut-Off
NINJ1 (pg/mL)	0.71 (0.63–0.80)	0.78	0.59	546.92
HMGB1 (ng/mL)	0.84 (0.77–0.90)	0.75	0.83	22.70
WBC (×10^9^)	0.63 (0.50–0.76)	0.31	0.37	14.34
CRP (mg/L)	0.58 (0.46–0.70)	0.56	0.22	93.81
ESR (mm/h)	0.82 (0.71–0.93)	0.75	0.8	38.90
Na (mmol/L)	0.69 (0.57–0.80)	0.5	0.14	136.50
PCT (ng/mL)	0.52 (0.39–0.66)	0.21	0.94	0.09
ALB (g/L)	0.60 (0.49–0.71)	0.19	0.63	37.05
LDH (U/L)	0.64 (0.54–0.74)	0.72	0.59	298
IL-1β (ng/mL)	0.46 (0.26–0.66)	0.62	0.59	4.85
IL-6 (ng/mL)	0.56 (0.40–0.71)	0.74	0.48	65.80
TNF-α (ng/mL)	0.53 (0.38–0.68)	0.68	0.47	2.45

NINJ1, Ninjurin-1; HMGB1, High Mobility Group Box 1; WBC, White blood cell; PCT, procalcitonin; ALB, Albumin; CRP, C-reactive protein; ESR, erythrocyte sedimentation rate; LDH, Lactate Dehydrogenase; IL-1β Interleukin-1β; IL-6, Interleukin-6; TNF-α, Tumor Necrosis Factor-alpha.

**Table 5:** ROC curve analysis results of various indicators for predicting coronary artery lesions (CALs) in children with Kawasaki Disease (KD).

Biomarker	AUC (95% CI)	Sensitivity	Specificity	Cut-Off
NINJ1 (pg/mL)	0.71 (0.61–0.82)	0.47	0.94	553.213
HMGB1 (ng/mL)	0.62 (0.50–0.74)	0.37	0.94	38.579
NINJ1 + CRP	0.74 (0.64–0.84)	0.74	0.71	-
WBC (×10^9^)	0.57 (0.43–0.70)	0.25	0.94	11.055
CRP (mg/L)	0.54 (0.39–0.68)	0.38	0.47	52.72
ESR (mm/h)	0.58 (0.41–0.76)	0.76	0.47	73.3
Na (mmol/L)	0.50 (0.36–0.64)	0.09	1	129.5
PCT (ng/mL)	0.55 (0.41–0.69)	0.52	0.24	0.804
ALB (g/L)	0.60 (0.44–0.75)	0.35	0.44	37.15
LDH (U/L)	0.65 (0.50–0.80)	0.67	0.61	356
IL-1β (ng/mL)	0.65 (0.50–0.80)	0.64	0.67	5.85
IL-6 (ng/mL)	0.49 (0.34–0.64)	0.65	0.25	97.3
TNF-α (ng/mL)	0.46 (0.28–0.65)	0.73	0.38	2.85

NINJ1, Ninjurin-1; HMGB1, High Mobility Group Box 1; WBC, White blood cell; PCT, procalcitonin; ALB, Albumin; CRP, C-reactive protein; ESR, erythrocyte sedimentation rate; LDH, Lactate Dehydrogenase; IL-1β Interleukin-1β; IL-6, Interleukin-6; TNF-α, Tumor Necrosis Factor-alpha.

## Data Availability

The original contributions presented in this study are included in the article/Supplementary Materials. Further inquiries can be directed to the corresponding authors.

## Supplementary Materials

The following supporting information can be downloaded at: https://www.mdpi.com/article/10.3390/biomedicines14020402/s1, Figure S1: Analysis of the correlation of serum NINJ1 and HMGB1 with disease day; Table S1: Exploratory correlational analyses of serum NINJ1 with inflammatory markers and cytokines, with False Discovery Rate (FDR) correction.

## Abbreviations

AAA	abdominal aortic aneurysm
ALB	Albumin
ALT	Aminotransferase
AST	Aspartate Aminotransferase
AUC	Area Under the Curve
CALs	coronary artery lesions
CI	Confidence Interval
CRP	C-reactive protein
Cx	circumflex
DAMPs	damage-associated molecular patterns
ELISA	enzyme-linked immunosorbent assay
ESR	Erythrocyte Sedimentation Rate
FC	febrile control
FDR	false discovery rate
Hb	Hemoglobin
HC	healthy control
HMGB1	High Mobility Group Box 1
IFN-α	Interferon-alpha
IL-1β	Interleukin-1β
IL-6	Interleukin-6
IVIG	intravenous immunoglobulin
KD	Kawasaki Disease
LAD	left anterior descending
LDH	Lactate Dehydrogenase
LMCA	left main coronary artery
Mono	Monocytes
nCALs	non-CALs
NINJ1	Ninjurin-1
PLT	Platelets
PCT	procalcitonin
RCA prox	Right Coronary Artery proximal
RCA dist	Right Coronary Artery distal
ROC	Receiver Operating Characteristic
TNF-α	Tumor Necrosis Factor-alpha
WBC	White blood cell
