# Supplementary material for: Changes in Serum Levels of NINJ1 and HMGB1 in Children with Kawasaki Disease and Their Clinical Significance
Source: Biomedicines. 2026 Feb 10;14(2):402. doi: 10.3390/biomedicines14020402 (PMC12938374; doi:10.3390/biomedicines14020402)
Supplement: Supplementary file 1 [file biomedicines-14-00402-s001.zip › biomedicines-4085455-supplementary.pdf]

Table S1. Exploratory correlational analyses of serum NINJ1 with inflammatory markers and cytokines, with False Discovery Rate (FDR) correction

Variable	Correlation Coefficient (r)	Raw *p*-Value	q-Value (FDR-Adjusted)	Significant (q < 0.05)
HMGB1	0.50	<0.001***	<0.001***	TRUE
TNF-α	0.19	0.05	0.39	FALSE
Hb	-0.13	0.19	0.65	FALSE
IL-6	-0.15	0.14	0.65	FALSE
LDH	0.11	0.24	0.65	FALSE
WBC	0.10	0.30	0.65	FALSE
ESR	0.11	0.27	0.65	FALSE
Monocyte	0.08	0.40	0.66	FALSE
CRP	0.09	0.37	0.66	FALSE
PLT	0.03	0.76	0.88	FALSE
IL-1β	0.04	0.75	0.88	FALSE
IFN-α	-0.04	0.75	0.88	FALSE
PCT	-0.05	0.63	0.88	FALSE
ALB	0.00	0.98	0.98	FALSE
ALT	-0.01	0.89	0.96	FALSE

NINJ1, Ninjurin-1; HMGB1, High Mobility Group Box 1; TNF-α, Tumor Necrosis Factor-alpha; Hb, Hemoglobin; IL-6, Interleukin-6; LDH, Lac tate Dehydrogenase; WBC, White blood cell; ESR, erythrocyte sedimentation rate; CRP, C-reactive protein; PLT, platelet; IL-1β Interleukin-1β; INF-α, Interferon-alpha; PCT, procalcitonin; ALB, Albumin; ALT Aminotransferase; $^{***}p<0.001$.

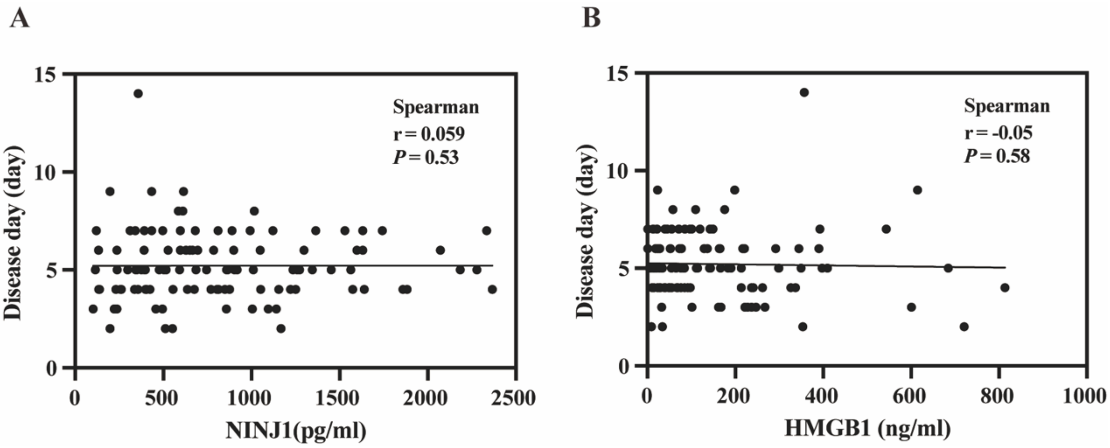

Figure S1: Analysis of the correlation of serum NINJ1 and HMGB1 with disease day. (A) No significant correlation was found between serum NINJ1 levels and disease day; (B) No significant correlation was found between serum HMGB1 levels and disease day; NINJ1, Ninjurin-1; HMGB1, High Mobility Group Box 1.
